# Cytokine Storms in COVID-19, Hemophagocytic Lymphohistiocytosis, and CAR-T Therapy

**DOI:** 10.1001/jamanetworkopen.2025.3455

**Published:** 2025-04-07

**Authors:** James P. Long, Rishab Prakash, Paul Edelkamp, Mark Knafl, Anath C. Lionel, Ranjit Nair, Sairah Ahmed, Paolo Strati, Luis E. Malpica Castillo, Ajlan Al-Zaki, Kelly Chien, Dai Chihara, Jason Westin, Fareed Khawaja, Loretta J. Nastoupil, Victor Mulanovich, Andrew Futreal, Scott E. Woodman, Naval G. Daver, Christopher R. Flowers, Sattva Neelapu, Joanna-Grace Manzano, Swaminathan P. Iyer

**Affiliations:** 1 Department of Biostatistics, The University of Texas MD Anderson Cancer Center, Houston; 2 Department of Lymphoma and Myeloma, Division of Cancer Medicine, The University of Texas MD Anderson Cancer Center, Houston; 3 Department of Enterprise Data Engineering & Analytics, The University of Texas MD Anderson Cancer Center, Houston; 4 Department of Leukemia, Division of Internal Medicine, The University of Texas MD Anderson Cancer Center, Houston; 5 Department of Infectious Diseases, The University of Texas MD Anderson Cancer Center, Houston; 6 Department of Genomic Medicine, The University of Texas MD Anderson Cancer Center, Houston; 7 Department of Hospital Medicine, Division of Internal Medicine, The University of Texas MD Anderson Cancer Center, Houston

## Abstract

**Importance:**

Cytokine storm (CS) is a hyperinflammatory syndrome causing multiorgan dysfunction and high mortality, especially in patients with malignant hematologic neoplasms. Triggers include malignant neoplasm–associated hemophagocytic lymphohistiocytosis (MN-HLH), cytokine release syndrome from chimeric antigen receptor T-cell therapy (CAR-T CRS), and COVID-19, but the underlying mechanisms of inflammation and their impact on outcomes are poorly understood.

**Objective:**

To delineate the inflammatory patterns characterizing different CS etiologies and their association with clinical outcomes.

**Design, Setting, and Participants:**

This retrospective cohort study was conducted at the MD Anderson Cancer Center in Houston, Texas, between March 1, 2020, and November 20, 2022, using the software-as-a-service Syntropy Foundry Platform. Participants were patients with malignant hematologic neoplasms who developed CS from COVID-19 (COVID-CS), MN-HLH, or CAR-T CRS.

**Exposure:**

Diagnostic criteria for COVID-CS were developed based on surging inflammatory markers (interleukin-6, C-reactive protein, and ferritin), while diagnosis of MN-HLH and CAR-T CRS followed established guidelines.

**Main Outcomes and Measures:**

The study compared cytokine levels, clinical characteristics, and survival outcomes across the 3 cohorts and focused on inflammatory markers, survival times, and key factors associated with survival identified through univariate and multivariable analyses.

**Results:**

A total of 671 patients met the inclusion criteria. Of those, 220 (33%) had CAR-T CRS, 227 (34%) had COVID-CS, and 224 (33%) had MN-HLH. Patients were predominantly male (435 [65%]), and 461 (69%) were White, with significant differences in median age (CAR-T CRS, 63 [IQR, 54-71] years; COVID-CS, 63 [IQR, 52-72] years; MN-HLH, 55 [IQR, 41-65] years; *P* < .001) as well as number of admission days and underlying cancer type across cohorts. Marked variations in cytokine levels and survival outcomes were observed, with the MN-HLH cohort exhibiting the highest levels of inflammatory markers (eg, median TNF-α, 105 pg/mL [IQR, 38-201 pg/mL] for MN-HLH vs 23 pg/mL [IQR, 17-42 pg/mL] for COVID-CS) and lowest fibrinogen and albumin levels. The cohort with CAR-T CRS showed substantially longer survival times compared with the cohort with COVID-CS (hazard ratio [HR], 2.93; 95% CI, 1.95-4.41) and the cohort with MN-HLH (HR, 8.12; 95% CI, 5.51-12.00). Clustering analysis showed overlapping patterns between COVID-CS and CAR-T CRS, while MN-HLH formed a distinct cluster.

**Conclusions and Relevance:**

This study of CS syndromes found distinct immune responses within each cohort. The distinct clinical patterns and outcomes associated with different CS etiologies emphasize the importance of early diagnosis and timely intervention.

## Key Points

**Question:**

How do clinical patterns and outcomes of cytokine storm (CS) vary among etiologies of cytokine release syndrome in chimeric antigen receptor T-cell therapy (CAR-T CRS), COVID-19 (COVID-CS), and malignant neoplasm–associated hemophagocytic lymphohistiocytosis (MN-HLH)?

**Findings:**

This cohort study of 671 patients with malignant hematologic neoplasms identified distinct inflammatory patterns and survival outcomes among the 3 cohorts with CS. Clustering analysis revealed overlapping patterns between COVID-CS and CAR-T CRS, while MN-HLH formed a separate cluster with the worst survival rates.

**Meaning:**

Understanding the unique and shared features of CS etiologies may inform personalized treatment strategies, highlighting the importance of early diagnosis and targeted interventions for improving patient outcomes.

## Teaser

This cohort study compares inflammatory patterns and clinical outcomes of cytokine storms in patients with COVID-19, patients receiving chimeric antigen receptor T-cell (CAR-T) therapy, and patients with malignant neoplasm–associated hemophagocytic lymphohistiocytosis.

## Introduction

One of the distinct features of COVID-19 caused by the β coronavirus SARS-CoV-2 is its ability to trigger a cascade of events that leads to a hyperinflammatory response characterized by activated macrophages and continuous release of cytokines. This exaggerated immune response has been linked to acute respiratory distress syndrome and potentially life-threatening complications.^1,2,3,4,5,6^ Patients with preexisting malignant hematologic neoplasms are particularly vulnerable to COVID-19–induced cytokine storm (COVID-CS), which has been associated with increased morbidity and mortality.^7,8,9^ However, the precise diagnostic criteria for COVID-CS are not fully defined.^10^ Furthermore, there are parallels among the CS observed in COVID-19, malignant neoplasm–associated hemophagocytic lymphohistiocytosis (MN-HLH), and chimeric antigen receptor T-cell (CAR-T) cytokine release syndrome (CRS), suggesting common underlying pathways.^11^ The lack of consensus on the definition, identification, and treatment of CS in COVID-19 makes it difficult to formally compare these conditions and understand the role and extent of dysregulated cytokine responses.

This study investigated the characteristics and outcomes of COVID-CS in patients with malignant hematologic neoplasms, the diagnostic criteria for identifying CS, and potential treatment strategies that may modulate the hyperinflammatory response. Elucidating the differences and similarities among these conditions can help develop treatment pathways to mitigate their severe consequences and improve patient outcomes.

## Methods

### Data Source

We conducted this retrospective cohort study comparing the characteristics and pathophysiology of 3 cohorts with CS (patients with COVID-19, MN-HLH, or CAR-T CRS) using the MD Anderson Context Engine, which is powered by the Syntropy Foundry Platform. This cloud-based, software-as-a-service platform integrates clinical, translational, and research data of all patients treated at MD Anderson Cancer Center. This enables extraction of insights from diverse data streams while maintaining security, compliance, and data governance. Data from consecutive patients were collected between March 1, 2020, and November 20, 2022, under the MD Anderson Data-Driven Determinants for COVID-19 Discovery Effort (D3CODE) institutional review board protocol. Informed consent was waived due to the retrospective nature of the study. The study followed the Strengthening the Reporting of Observational Studies in Epidemiology (STROBE) reporting guideline.

### Study Population

The study included patients with malignant hematologic neoplasms diagnosed with COVID-19 and MN-HLH and patients with CRS following standard-of-care CAR-T therapy for lymphoma (axicabtagene ciloleucel, lisocabtagene maraleucel, tisagenlecleucel, or brexucabtagene autoleucel) or myeloma (idecabtagene vicleucel or ciltacabtagene autoleucel). All included patients were admitted to our institution during the study period.

### Data Collection

The study collected data on demographics, clinical presentation, laboratory parameters, treatments, outcomes, and levels of inflammatory markers, including interleukin 6 (IL-6), soluble IL-2 receptor (sIL-2R), ferritin, C-reactive protein (CRP), and lactate dehydrogenase (LDH). Other laboratory values analyzed included alanine aminotransferase (ALT), alkaline phosphatase (ALP), bilirubin (direct and indirect), creatinine, D-dimer, fibrinogen, white blood cell (WBC) count, absolute lymphocyte count, absolute monocyte count, absolute neutrophil count, platelet count, tumor necrosis factor (TNF) α, troponin T, and venous lactate.

Race, ascertained by self-report, was included in the analysis to reduce any confounding potential of immunological and genetic variations due to race. Categories were Asian, Black or African American, White, and other (included American Indian or Alaska Native and those who declined to answer).

### Definition of COVID-CS, CAR-T CRS, and MN-HLH

Using *International Statistical Classification of Diseases and Related Health Problems, 10th Revision (ICD-10)* codes, we identified confirmed SARS-CoV-2 infections at our institution. We then selected patients with COVID-19–associated hyperinflammatory syndrome based on the scoring system of Webb et al;^12^ our method included the common inflammatory markers ferritin, CRP, and IL-6. The COVID-CS diagnosis date was defined as the date when the latter of these laboratory values exceeded the upper limit of normal (within 28 days of confirmed SARS-CoV-2 infection). The flow diagram in eFigure 1 in Supplement 1 describes the selection of patients with CS. Patients who met 2 or more COVID-CS criteria were defined as having COVID-CS; those not meeting criteria were defined as COVID-CS negative. Patients with solid tumors were excluded. The CAR-T cohort was identified using *Current Procedural Technology* code 0540T and *ICD-10* code D89.83 for CRS, as defined by the American Society for Transplantation and Cellular Therapy consensus grading.^13^ MN-HLH cases were identified by *ICD-10* code D76.1 and confirmed when they met the HLH-2004 criteria.^14^ In addition, we conducted medical record reviews, when available.

### Survival Models by Cohort

For the cohort with CAR-T CRS, the survival time was calculated from the earliest infusion date (baseline) to the date of death. For the cohorts with COVID-CS and MN-HLH, the survival time was calculated from the earliest date of laboratory testing that defined COVID-CS and MN-HLH (baseline) to the date of death. In all cohorts, patients alive as of November 16, 2022, were censored at that date.

### Statistical Analysis

We used descriptive statistics to summarize demographic and clinical characteristics, Kruskal-Wallis rank sum test to compare continuous variable distributions, and χ^2^ or Fisher exact test to compare categorical variables. Heat maps were used to visualize laboratory value distributions. We used the default parameter setting of the ComplexHeatmap package (hierarchical agglomerative clustering with Euclidean distance metric and complete linkage) in R, version 4.2 (R Project for Statistical Computing) to identify distinct sets of laboratory values and patients in the heat map.^15^ Kaplan-Meier curves, log-rank tests, Cox proportional hazards regression models, and random survival forests (RSFs) were used to compare survival outcomes. For Cox proportional hazards regression models, variables were normalized prior to fitting. RSFs were used to avoid the proportional hazards assumption made by Cox proportional hazards regression models. Permutation variable importance was used to assess the importance of each prognostic variable in RSF models.^16,17^ A 2-sided *P* < .05 was considered statistically significant. We used *t*-distributed stochastic neighbor embedding (*t*-SNE) to visualize biomarker distribution across cohorts.^18^ For multivariate analyses, missing values were imputed using the median. For univariate regression models, observations with missing values were excluded. Statistical analyses were performed in R, version 4.1.1, with packages gtsummary, ggplot2, survival, survminer, ComplexHeatmap, Rtne, and randomForestSRC.^15,19,20,21,22,23^

## Results

### Characteristics of the Cohort With COVID-CS

We identified 9032 patients with a confirmed SARS-CoV-2 infection at our institution. Our analysis revealed marked differences between the group with COVID-CS (n = 337) and the group that was COVID-CS negative (n = 8695) across multiple laboratory parameters (eTable 1 in Supplement 1). Significant differences were observed in ALT level, ALP level, bilirubin (direct and indirect) level, creatinine level, D-dimer level, fibrinogen level, LDH level, WBC count, absolute lymphocyte count, absolute monocyte count, absolute neutrophil count, platelet count, tumor necrosis factor (TNF)–α level, troponin T level, and venous lactate level. A heat map analysis revealed distinct laboratory profiles distinguishing patients with COVID-CS from COVID-CS–negative patients across multiple parameters. Importantly, patients with COVID-CS had significantly worse survival compared with COVID-CS–negative patients (eFigure 2 in Supplement 1).

### Comparison of the 3 Cohorts With CS

#### Baseline Clinical Features

We analyzed 671 patients with hematologic neoplasms (236 [35%] female; 435 [65%], male). Thirty patients (4%) were Asian; 87 (13%), Black or African American; 461 (69%), White; and 89 (13%), other race (race was not reported for 3 patients in the COVID-CS and 1 in the MN-HLH cohort). A total of 220 patients (33%) developed CAR-T CRS; 227 (34%), COVID-CS; and 224 (33%), MN-HLH. The demographic and clinical characteristics of the 3 cohorts are compared in Table 1. Sex distribution among the 3 cohorts was comparable. The group with CAR-T CRS had a higher proportion of White patients (165 [75%]) compared with the cohorts with COVID-CS (149 [67%]) and MN-HLH (147 [66%]) and a lower percentage of Black or African American patients (20 [9%]) compared with the groups with COVID-CS (36 [16%]) and MN-MLH (31 [14%]). The group with MN-HLH had a lower median age than other cohorts (55 years [IQR, 41-65 years]), compared with 63 years (IQR, 54-71 years) in the CAR-T CRS and 63 years (IQR, 52-72 years) in the COVID-CS cohort (*P* < .001). Significant differences were observed in cancer type across the cohorts. The cohort with CAR-T CRS had a higher proportion of diffuse large B-cell lymphoma (136 [68%] vs 54 [24%] for COVID-CS and 69 [34%] for MN-MLH). In contrast, leukemia was more prevalent in the groups with COVID-CS (102 [45%]) and MN-HLH (85 [41%]) compared with the group with CAR-T CRS (2 [1%]). Additionally, T-cell lymphoma was more prevalent in the group with MN-HLH (26 [13%]) than in the group with COVID-CS (9 [4%]), and no patients with CAR-T CRS had T-cell lymphoma. Fever was common in all cohorts (≥89%) but highest in the group with CAR-T CRS (216 patients [98%]). Median number of admission days was greater in the group with MN-HLH (24; IQR, 12-38) than in the groups with COVID-CS (6; IQR, 4-9) and CAR-T CRS (15; IQR, 11-21). In the cohort with COVID-CS, 158 patients (70%) received remdesivir. In the cohort with COVID-CS, 80 patients (35%) were fully vaccinated, 19 (8%) partially vaccinated, and 128 (56%) had unknown vaccination status (a detailed analysis is in Chien et al^24^). In addition, in the group with CAR-T CRS, tocilizumab was administered with greater frequency (154 patients [70%]) than in the groups with COVID-CS (79 [35%]) and MN-HLH (102 [46%]). Race data were missing for 4 patients (1%), admission days for 120 (18%), and cancer type for 39 (6%). Other variables had no missing data.

**Table 1.** Baseline Clinical Characteristics

Characteristic	Patients (N = 671)^a^			*P* value^b^
	CAR-T CRS (n = 220)	COVID-CS (n = 227)	MN-HLH (n = 224)	
Gender				
Female	69 (31)	77 (34)	90 (40)	.14
Male	151 (69)	150 (66)	134 (60)	
Race^c^				
Asian	11 (5)	4 (2)	15 (7)	.03
Black or African American	20 (9)	36 (16)	31 (14)	
White	165 (75)	149 (67)	147 (66)	
Other^d^	24 (11)	35 (16)	30 (13)	
Age, median (IQR), y	63 (54-71)	63 (52-72)	55 (41-65)	<.001
Length of stay, median (IQR), d	15 (11-21)	6 (4-9)	24 (12-38)	<.001
ICU at baseline	48 (22)	20 (9)	104 (46)	<.001
Received mechanical ventilation	10 (5)	44 (19)	61 (27)	<.001
Vasopressor use	18 (8)	51 (22)	82 (37)	<.001
Tocilizumab use	154 (70)	79 (35)	102 (46)	<.001
COVID-19 vaccination				
Complete	110 (50)	80 (35)	24 (11)	<.001
Incomplete	26 (12)	19 (8)	9 (4)	
Unknown	84 (38)	128 (56)	191(85)	
Fever	216 (98)	202 (89)	208 (93)	<.001
Cancer type				
DLBCL	136 (68)	54 (24)	69 (34)	<.001
Hodgkin lymphoma	0	2 (1)	8 (4)	
Follicular lymphoma	22 (11)	28 (12)	11 (5)	
Leukemia	2 (1)	102 (45)	85 (41)	
Mantle cell lymphoma	16 (8)	6 (3)	0	
Myeloma	24 (12)	26 (11)	6 (3)	
T-cell lymphoma	0	9 (4)	26 (13)	

Abbreviations: CAR-T CRS, chimeric antigen receptor T-cell cytokine release syndrome; COVID-CS, COVID-19 cytokine storm; DLBCL, diffuse large B-cell lymphoma; ICU, intensive care unit; MN-HLH, malignant neoplasm–associated hemophagocytic lymphohistiocytosis.

^a^ Data are presented as number (percentage) of participants unless otherwise indicated. Race data were missing for 4 patients (1%), admission days for 120 (18%), and cancer type for 39 (6%).

^b^ Fisher exact test for count data with simulated *P* value (based on 2000 replicates); Kruskal-Wallis rank sum test.

^c^ Race was not reported for 3 patients in the COVID-CS and 1 in the MN-HLH cohort.

^d^ Included American Indian or Alaska Native and those who declined to answer.

#### Baseline Laboratory Features

**Figure 1.**
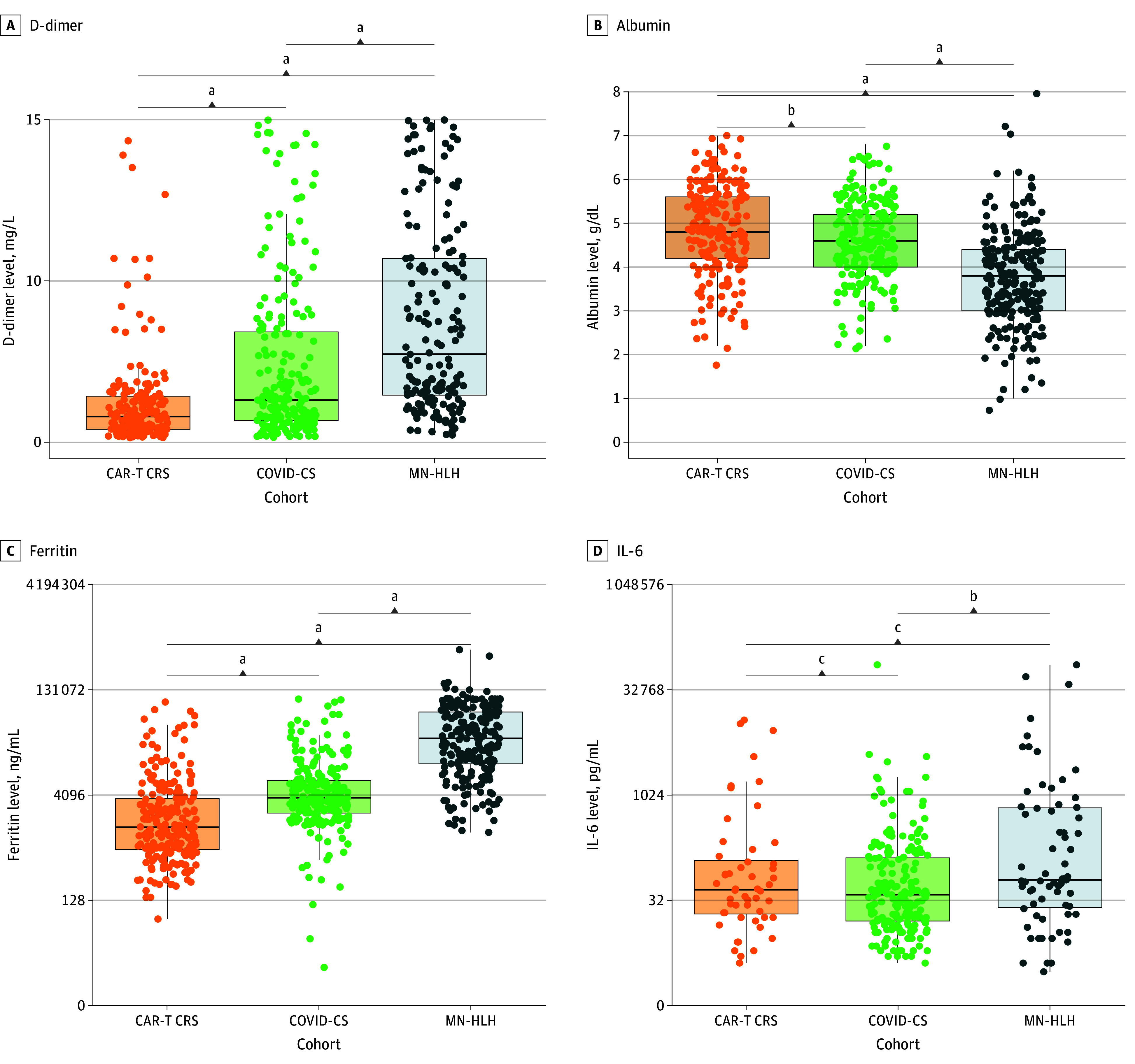
Box Plots Showing Differences in Laboratory Values by Cytokine Storm Etiology Circles represent individual laboratory values; the horizontal bar inside the boxes, the median; and the lower and upper ends of the boxes, the first and third quartiles. Whiskers extend from the median up to the lesser of the maximum values and 1.5 times the IQR and down to the greater of the minimum values and 1.5 times the IQR. CAR-T CRS indicates chimeric antigen receptor T-cell cytokine release syndrome; COVID-CS, COVID-19 cytokine storm; IL-6, interleukin 6; MN-HLH, malignant neoplasm–associated hemophagocytic lymphohistiocytosis. ^a^*P* < .001. ^b^*P* < .01. ^c^No significant difference.

Table 2 compares various laboratory parameters among the 3 cohorts. For hematologic laboratory results (Figure 1), the cohort with MN-HLH had lower values of WBCs, absolute neutrophils, absolute monocytes, hemoglobin, platelets, and fibrinogen compared with the cohorts with CAR-T CRS and COVID-CS. The cohort with MN-HLH exhibited an increase in D-dimer level. CRP levels exceeded the established normal threshold of 5 mg/dL in all cohorts (medians ranged from 136 mg/dL [IQR, 56-213 mg/dL] for MN-MLH to 147 mg/dL [IQR, 93-226 mg/dL] for COVID-CS) (to convert CRP to mg/L, multiply by 10); however, no significant variations were observed across cohorts. In contrast, the erythrocyte sedimentation rate was higher in the cohort with COVID-CS than in the cohorts with CAR-T CRS and MN-HLH. Significant differences between cohorts were also observed in other inflammatory markers: ferritin, LDH, sIL-2R, IL-6, and TNF-α. Patients with MN-HLH exhibited substantially higher levels of total bilirubin, direct bilirubin, indirect bilirubin, and ALT. Albumin levels were considerably lower in patients with MN-HLH. In addition, laboratory values for creatinine, troponin T, and venous lactate were higher in patients with MN-HLH. eTable 2 in Supplement 1 summarizes the missingness of the laboratory values. Of the 25 laboratory values considered, 14 (56%) had data missing for less than 5% of patients.

**Table 2.** Baseline Laboratory Parameters

Parameter	Laboratory value, median (IQR)			*P* value^a^
	CAR-T CRS (n = 220)	COVID-CS (n = 227)	MN-HLH (n = 224)	
WBC count, /μL	400 (200-900)	1600 (500-3400)	100 (0-700)	<.001
Absolute neutrophil count, /μL	6000 (4000-11 000)	6000 (3000-11 000)	5000 (2000-9000)	.007
Absolute lymphocyte count, /μL	880 (510-1540)	910 (540-1630)	790 (380-1780)	.50
Absolute monocyte count, /μL	820 (540-1330)	730 (380-1250)	610 (200-1200)	.003
Hemoglobin, g/dL	7.70 (7.10-8.90)	7.30 (6.60-9.10)	6.80 (6.10-7.20)	<.001
Platelet count, ×10^3^/μL	40 (13-73)	41 (11-105)	6 (3-12)	<.001
Fibrinogen, mg/dL	255 (174-348)	322 (220-445)	166 (93-312)	<.001
D-dimer, μg/mL FEU	1.6 (0.8-2.9)	2.6 (1.4-6.8)	5.5 (2.9-11.4)	<.001
CRP, mg/dL	140 (81-198)	147 (93-226)	136 (56-213)	.12
ESR, mm/h	35 (16-58)	84 (64-110)	53 (30-92)	<.001
Ferritin, ng/mL	1420 (683-3637)	3742 (2262-6572)	26 386 (11 430-63 043)	<.001
LDH, U/L	307 (248-431)	414 (280-636)	1166 (623-3877)	<.001
IL-6, pg/mL	45 (20-118)	38 (16-128)	62 (25-667)	.008
sIL-2R, pg/mL	24 456 (6487-50 808)	2609 (1616-7448)	8194 (3579-29 240)	.003
TNF-α, pg/mL	32 (20-81)	23 (17-42)	105 (38-201)	<.001
ALT, U/L	46 (30-86)	45 (27-81)	144 (67-318)	<.001
ALP, U/L	107 (88-146)	113 (87-157)	301 (166-541)	<.001
Bilirubin, mg/dL				
Total	0.9 (0.6-1.3)	0.8 (0.6-1.3)	2.3 (1.2-6.0)	<.001
Direct	0.3 (0.2-0.4)	0.3 (0.2-0.5)	1.6 (0.5-4.7)	<.001
Indirect	0.7 (0.5-1.0)	0.6 (0.4-0.9)	1.0 (0.7-1.6)	<.001
Albumin, g/dL	2.90 (2.60-3.30)	2.80 (2.50-3.10)	2.40 (2.00-2.70)	<.001
Creatinine, mg/dL	1.08 (0.90-1.39)	1.12 (0.85-1.47)	1.29 (0.87-2.57)	.001
Troponin T, ng/mL	0.02 (0.13-0.14)	0.29 (0.15-0.63)	0.40 (0.21-0.93)	<.001
Sodium, mEq/L	133.0 (131.8-135.3)	133.0 (131.0-135.0)	132.0 (128.0-134.0)	<.001
Venous plasma lactate, mg/dL	108.11 (81.08-171.17)	126.13 (90.09-180.18)	243.24 (164.86-421.62)	<.001

Abbreviations: ALP, alkaline phosphatase; ALT, alanine transaminase; CAR-T CRS, chimeric antigen receptor T-cell cytokine release syndrome; COVID-CS, COVID-19 cytokine storm; CRP, C-reactive protein; ESR, erythrocyte sedimentation rate; FEU, fibrinogen equivalent units; sIL-2R, soluble interleukin 2 receptor; IL-6, interleukin 6; LDH, lactate dehydrogenase; MN-HLH, malignant neoplasm–associated hemophagocytic lymphohistiocytosis; TNF, tumor necrosis factor; WBC, white blood cell.

SI conversion factors: To convert albumin to g/L, multiply by 10; ALP to μkat/L, multiply by 0.0167; ALT to μkat/L, multiply by 0.0167; creatinine to μmol/L, multiply by 88.4; CRP to mg/L, multiply by 10; direct bilirubin to μmol/L, multiply by 17.104; D-dimer to nmol/L, multiply by 5.476; ESR to mm/h, multiply by 1.0; ferritin to μg/L, multiply by 1.0; fibrinogen to g/L, multiply by 0.01; hemoglobin to g/L, multiply by 10.0; lactate to mmol/L, multiply by 0.111; LDH to μkat/L, multiply by 0.0167; lymphocytes to ×10^9^/L, multiply by 0.001; monocytes to ×10^9^/L, multiply by 0.001; neutrophils to ×10^9^/L, multiply by 0.001; plasma lactate to mmol/L, multiply by 0.111; platelet count to ×10^9^/L, multiply by 1.0; sodium to mmol/dL, multiply by 1.0; total bilirubin to μmol/L, multiply by 17.104; troponin T to μg/L, multiply by 1.0; WBC count to ×10^9^/L, multiply by 0.001.

^a^ Kruskal-Wallis rank sum test.

#### Association Between CS Features and Disease Etiology

**Figure 2.**
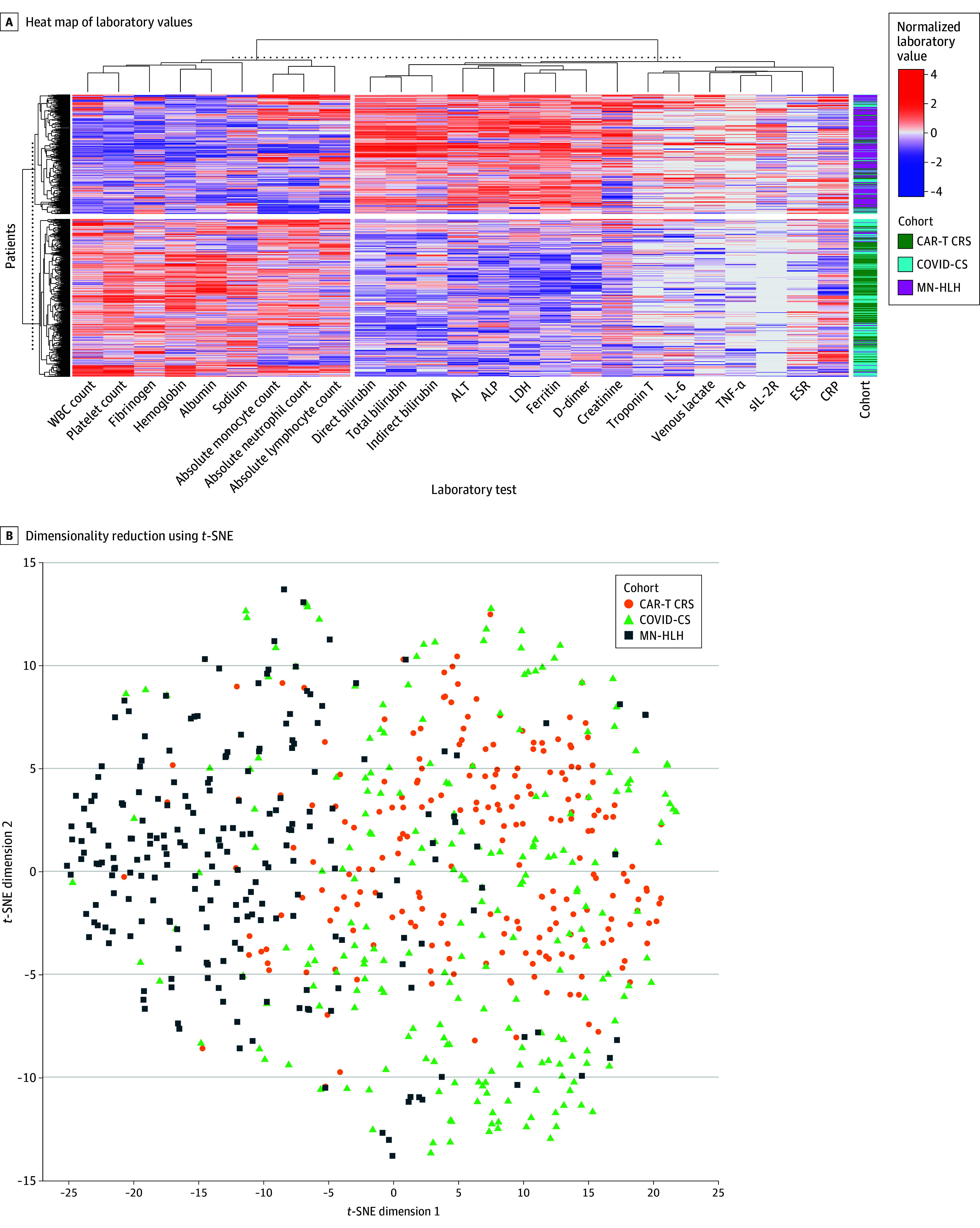
Heat Map of Laboratory Values and 2-Dimensional Reduction for the 3 Patient Cohorts With Cytokine Storm Etiology A, Laboratory values (columns) and patients (rows) are clustered. Brackets represent the clustering found in the hierarchical clustering model. Data were transformed using quartile normalization. ALP indicates alkaline phosphatase; ALT, alanine transaminase; CAR-T CRS, chimeric antigen receptor T-cell cytokine release syndrome; COVID-CS, COVID-19 cytokine storm; CRP, C-reactive protein; ESR, erythrocyte sedimentation rate; IL-6, interleukin 6; LDH, lactate dehydrogenase; MN-HLH, malignant neoplasm–associated hemophagocytic lymphohistiocytosis; sIL-2R, soluble interleukin 2 receptor; TNF, tumor necrosis factor; *t*-SNE, *t*-distributed stochastic neighbor embedding; WBC, white blood cell.

The heat map of continuous laboratory values illustrates 2 distinct CS patterns for the cohorts (Figure 2A). The first patient cluster was highly enriched for MN-HLH, whereas the second patient cluster was a mixture of patients with COVID-CS and CAR-T CRS. The first laboratory cluster, including WBC, platelet, and absolute monocyte, neutrophil, and lymphocyte counts as well as fibrinogen level, was low in the MN-HLH cluster and high in the COVID-CS and CAR-T CRS clusters. The reverse trend was observed in the second laboratory cluster, including total, indirect, and direct bilirubin; venous lactate; TNF-α; sIL-2R; troponin T; IL-6; LDH; ferritin; D-dimer; ALT; ALP; and creatinine levels. Overall, by clustering analysis, COVID-CS was more similar to CAR-T CRS than MN-HLH–induced CS and MN-HLH CS was more severe than CAR-T CRS and COVID-CS. The first 2 dimensions of a *t*-SNE plot of the laboratory values (Figure 2B) show that MN-HLH was considerably distinct from CAR-T CRS and COVID-CS. The latter 2 cohorts had substantial similarities, with only marginal differences between them. To assess whether differences in laboratory parameters could be associated with baseline factors (eg, age, race) rather than disease etiology, we fit regression models of laboratory values by disease etiology, controlling for baseline confounders (eTable 3 in Supplement 1). Differences in laboratory parameters remained significant, suggesting that differences were associated with disease etiology, not baseline confounders.

### Survival Characteristics

**Figure 3.**
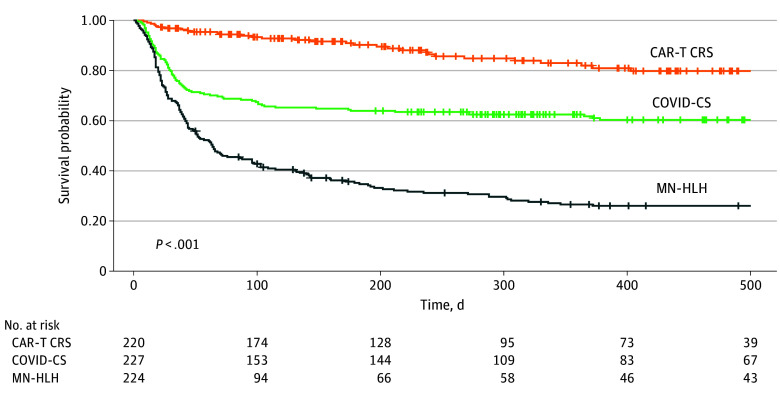
Overall Survival Among the 3 Cytokine Storm Cohorts Vertical hash marks represent censoring. CAR-T CRS indicates chimeric antigen receptor T-cell cytokine release syndrome; COVID-CS, COVID-19 cytokine storm; MN-HLH, malignant neoplasm–associated hemophagocytic lymphohistiocytosis.

Long-term survival probabilities varied considerably among cohorts with CS (Figure 3). The group with CAR-T CRS had the best survival. Conversely, the survival rate among patients with MN-HLH and COVID-CS was significantly lower. Patients with MN-HLH also had a faster decline than those with COVID-CS, as illustrated by the steep gradient of the MN-HLH survival curve. The 28-day probability of survival was 97% (95% CI, 95%-99%) for CAR-T CRS, 81% (95% CI, 76%-86%) for COVID-CS, and 75% (95% CI, 63%-75%) for MN-HLH. The 100-day probability of survival was 93% (95% CI, 90%-97%) for CAR-T CRS, 67% (95% CI, 62%-74%) for COVID-CS, and 42% (95% CI, 37%-50%) for MN-HLH. Additionally, a Cox proportional hazards regression model was fit to the data, controlling for race, gender, and age (eTable 4 in Supplement 1). The hazard ratios for COVID-CS and MN-HLH (compared with CAR-T CRS) were 2.93 (95% CI, 1.95-4.41) and 8.12 (95% CI, 5.51-12.00), respectively, indicating that significantly lower survival in these cohorts persisted even after controlling for demographic variables. The age confounder was also substantial, with lower survival with increasing age. Survival differences by race and gender were not statistically significant. In addition, patients with COVID-CS receiving tocilizumab had worse survival (eFigure 3 in Supplement 1). In the cohorts with CAR-T CRS and MN-HLH, there were no significant differences between those who received and did not receive tocilizumab.

### Factors Associated With Overall Survival

For each laboratory and demographic variable, we fit univariate Cox proportional hazards regression models^25^ (eTable 5 in Supplement 1). Many laboratory values were significantly associated with survival. Ferritin and LDH level had the highest HRs and platelet count, the lowest. Next, we fit multivariable Cox proportional hazards regression models and RSFs using all laboratory and demographic variables and cohorts. RSF measures showed that ferritin level, LDH level, and platelet count were the most important variables, followed by cohort (eFigure 4 in Supplement 1). The Cox proportional hazards regression model yielded similar qualitative outcomes, wherein the cohort variable was significant (eTable 6 in Supplement 1).

## Discussion

The severity of COVID-19 is influenced by viral variants and host factors, such as age, comorbidities, and evolving immunologic responses.^1,2^ A critical aspect in the postviremic phase is the dysregulation of the immune response, characterized by excessive release of inflammatory mediators leading to a CS.^3,4,5,6^ Timely diagnosis and treatment require identifying this inflammatory component. Although diagnostic and therapeutic protocols are established for CS in conditions like MN-HLH and CAR-T CRS, no studies to our knowledge have determined clinical parameters unique to COVID-CS.^7,9,13,14^ This gap underscores the need to establish standardized criteria for defining COVID-CS.^10,11,12^ While Webb et al^12^ recommended using a COVID-19–associated hyperinflammatory syndrome scoring system that incorporates clinical and laboratory parameters (6 categories), we developed a simplified method using common inflammatory markers to define COVID-CS. This simplification was necessary to avoid overlap between symptoms of malignant hematologic neoplasms and the CS, to enable earlier detection, and to create a more targeted definition for a unique patient population. This approach distinguished patients who had COVID-CS from COVID-CS–negative patients across multiple laboratory parameters. Additionally, patients with COVID-CS showed significantly worse survival rates, validating our classification method and highlighting the clinical relevance of these laboratory distinctions.^10,11,12^

Furthermore, our comparison of clinical presentation, laboratory parameters, and outcomes across cohorts with COVID-CS, CAR-T CRS, and MN-HLH revealed several key differences. The cohorts with COVID-CS and MN-HLH showed higher proportions of leukemia and lymphoma, including T-cell lymphoma, compared with the cohort with CAR-T CRS. While fever incidence was consistent across cohorts (highest in CAR-T CRS), patients with MN-HLH had the longest hospital stay (median of 24 days) and different patterns of tocilizumab administration. The variation in laboratory parameters among the cohorts exhibited distinctive trends. Patients with MN-HLH showed lower WBC, neutrophil, monocyte, and platelet counts and hemoglobin levels, which is consistent with immune cell infiltration in organs and cytokine-mediated suppression of hematopoiesis in HLH. All cohorts had coagulation abnormalities; however, the fibrinogen level was elevated in the cohort with COVID-CS but significantly decreased in the cohort with MN-HLH.

Elevated ferritin levels were a hallmark finding, although the extent of elevation differed among the cohorts. CRP levels were similar in COVID-CS, MN-HLH, and CAR-T CRS. By clustering analysis, COVID-CS was more similar to CAR-T CRS than MN-HLH.^26,27,28,29^ The distinct pattern of inflammatory parameters points to unique pathophysiological processes. These inflammatory markers also serve as indicators of immune-mediated endothelial toxic effects in patients receiving CAR-T. This relationship is exemplified by the Endothelial Activation and Stress Index, Ferritin, and CRP score, which provides a comprehensive measure of endothelial dysfunction and systemic inflammation.^30,31^

The trajectory of CS was significantly associated with patient outcomes, with different cohorts with CS showing different survival probabilities over time.^26,28,29^ The cohort with CAR-T had the highest survival probability followed by the groups with COVID-CS and MN-HLH. Cox proportional hazards regression models and multivariate analyses revealed that the CS types remained significantly associated with survival even after controlling for demographic variables and laboratory values. Regression analysis adjusting for confounders revealed that disease etiology was significantly associated with survival both directly and indirectly. The indirect associations in this study were mediated through inflammatory markers, like ferritin and LDH levels, while direct associations were likely attributable to unmeasured factors, such as cohort-specific immune responses. This dual impact underscores the complex relationship between disease origin and patient outcomes.

While the general definition of CS applies across various etiologies, there were differences in manifestations, cytokine signatures, and management strategies affecting the clinical course. These variations stem from distinct underlying mechanisms, including viral-induced dysregulation in COVID-CS,^24^ massive T-cell activation in CAR-T CRS,^32^ and impaired natural killer and cytotoxic T-cell function in MN-HLH.^33,34,35^ Second, the host immune status varies across these conditions. Patients with hematologic neoplasms have dysregulated immunity at baseline, patients with CAR-T undergo lymphodepletion before cell infusion, and patients with COVID-19 have variable degrees of immune compromise.^24^ Third, the kinetics of CS development differ among these etiologies. CAR-T CRS has a more predictable early onset; in contrast, COVID-CS and MN-HLH have a subacute presentation, delaying recognition and treatment.^33,34,35^ Fourth, treatment approaches are tailored to each condition. In CAR-T CRS, the management approach may be adjusted based on the severity of the CS. The protocolized use of tocilizumab and steroids improves outcomes.^24^ Immunosuppressive agents like dexamethasone or etoposide are used in MN-HLH to control excessive immune activation,^33,34,35^ while emerging biologics like anti-interferon-γ monoclonal antibodies are being explored.^36^ In COVID-19, antiviral therapies and corticosteroids have shown efficacy in reducing mortality rates.^37^ Anakinra targeting IL-1 and IL-6 has shown promise in improving survival and shortening hospital stays in patients with elevated soluble urokinase plasminogen activator receptor levels.^38,39,40^ In our study, the lack of association between tocilizumab and survival may be due to selection of patients with advanced-stage CS and poor outcomes, highlighting the complexity of managing these conditions.

### Strengths and Limitations

The main strength of the study is in using large, curated patient cohorts and focusing on hematologic neoplasms to analyze the pathophysiology and treatment of CS. The Syntropy Foundry Platform enabled seamless data consolidation and the revelation of patterns and associations that were previously hidden. The study also differentiated COVID-CS, defined using common inflammatory markers, from MN-HLH and CAR-T CRS based on survival probability and laboratory values.

This study also has limitations. The retrospective design limits causal inferences about the effects of disease etiology on laboratory values and outcomes. The lack of consistent inflammatory marker measurements may underestimate CS prevalence in patients with COVID-19. Furthermore, the evolving nature of COVID-19 treatment protocols during the study period may have influenced the results. We did not analyze Janus kinase inhibitor use or examine in detail the association of COVID-19 vaccination status with CS outcomes. In the cohort with CAR-T, the predominant use of axicabtagene ciloleucel, which uses the CD28 costimulatory domain, may have been associated with a higher rate of CRS. Additionally, the cohort with MN-HLH had a higher incidence of T-cell lymphoma, possibly contributing to increased mortality. Our single-center findings also warrant validation in external cohorts to ensure broader applicability. Despite these limitations, our study advances the understanding of CS pathophysiology and treatment, underscoring the need for timely interventions.

## Conclusions

This cohort study identified COVID-CS by using common inflammation markers and compared it with 2 other CS conditions: MN-HLH and the CRS in CAR-T. The CAR-T–treated group exhibited the highest survival outcomes compared with the cohorts with COVID-CS and MN-HLH. Additionally, the findings suggest that CAR-T CRS and COVID-CS share similar cytokine activation pathways, while MN-HLH operates differently. The current treatments targeting cytokines are inadequate for COVID-CS and MN-HLH, highlighting the need for clinical trials for new pathway inhibitors. Furthermore, understanding the enduring effects of COVID-CS is vital, as they may be linked to post-COVID condition symptoms. Overall, the study underscores the need for further investigations into treatment strategies for CS in patients with hematologic neoplasms.

## Notes

Supplement 1. **eTable 1.** Comparison of COVID-CS and COVID-CS–Negative Cohorts **eTable 2.** Summary of Missingness **eTable 3.** Regression Model of Laboratory Values by Cause of CS **eTable 4.** Cox Proportional Hazards Regression of Survival by Demographic Variables and Cohort **eTable 5.** Univariate Cox Proportional Hazards Regression of Laboratory Values Comparing CAR-T CRS, COVID-CS, and MA-HLH Cohorts **eTable 6.** Multivariate Cox Proportional Hazards Regression of Laboratory Values Comparing CAR-T CRS, COVID-CS, and MA-HLH Cohorts **eFigure 1.** Flow Diagram **eFigure 2.** Comparison of Survival Between Patients With COVID-19 Diagnosed and Not Diagnosed With Cytokine Storm **eFigure 3.** Association of Tocilizumab With Overall Survival by Cohort **eFigure 4.** Variable Importance in the Random Survival Forest Model

Supplement 2.
Data-Driven Determinants for COVID-19 Discovery Effort (D3CODE) Team

Supplement 3.
Data Sharing Statement
