# Error in the Conflict of Interest Disclosures

**DOI:** 10.1001/jamanetworkopen.2025.57445

**Published:** 2026-01-15

In the Original Investigation titled “Cytokine Storms in COVID-19, Hemophagocytic Lymphohistiocytosis, and CAR-T Therapy,” published on April 7, 2025, in the Conflict of Interest Disclosures, the grant number RR190079 was added for Cancer Prevention and Research Institute of Texas Scholar in Cancer Research for Dr Flowers. This article has been corrected online.^1^
