# Supplementary material for: Cytokine Storms in COVID-19, Hemophagocytic Lymphohistiocytosis, and CAR-T Therapy
Source: JAMA Netw Open. 2025 Apr 7;8(4):e253455. doi: 10.1001/jamanetworkopen.2025.3455 (PMC11976493; doi:10.1001/jamanetworkopen.2025.3455)
Supplement: Supplement 1. — eTable 1. Comparison of COVID-CS and COVID-CS–Negative Cohorts eTable 2. Summary of Missingness eTable 3. Regression Model of Laboratory Values by Cause of CS eTable 4. Cox Proportional Hazards Regression of Survival by Demographic Variables and Cohort eTable 5. Univariate Cox Proportional Hazards Regression of Laboratory Values Comparing CAR-T CRS, COVID-CS, and MA-HLH Cohorts eTable 6. Multivariate Cox Proportional Hazards Regression of Laboratory Values Comparing CAR-T CRS, COVID-CS, and MA-HLH Cohorts eFigure 1. Flow Diagram eFigure 2. Comparison of Survival Between Patients With COVID-19 Diagnosed and Not Diagnosed With Cytokine Storm eFigure 3. Association of Tocilizumab With Overall Survival by Cohort eFigure 4. Variable Importance in the Random Survival Forest Model [file jamanetwopen-e253455-s001.pdf]

Supplementary Online Content

Long JP, Prakash R, Edelkamp P Jr, et al; Data-Driven Determinants for COVID-19 Discovery Effort (D3CODE) Team. Cytokine storms in COVID-19, hemophagocytic lymphohistiocytosis, and CAR-T therapy. *JAMA Netw Open*. 2025;8(4):e253455. doi:10.1001/jamanetworkopen.2025.3455

eTable 1. Comparison of COVID-CS and COVID-CS–Negative Cohorts
eTable 2. Summary of Missingness
eTable 3. Regression Model of Laboratory Values by Cause of CS
eTable 4. Cox Proportional Hazards Regression of Survival by Demographic Variables and Cohort
eTable 5. Univariate Cox Proportional Hazards Regression of Laboratory Values Comparing CAR-T CRS, COVID-CS, and MA-HLH Cohorts
eTable 6. Multivariate Cox Proportional Hazards Regression of Laboratory Values Comparing CAR-T CRS, COVID-CS, and MA-HLH Cohorts
eFigure 1. Flow Diagram
eFigure 2. Comparison of Survival Between Patients With COVID-19 Diagnosed and Not Diagnosed With Cytokine Storm
eFigure 3. Association of Tocilizumab With Overall Survival by Cohort
eFigure 4. Variable Importance in the Random Survival Forest Model

This supplementary material has been provided by the authors to give readers additional information about their work.

© 2025 Long JP et al. *JAMA Network Open*.

eTable 1: Comparison of COVID-CS and COVID-CS Neg cohorts.

Characteristic	**COVID-CS**, N = 337	**COVID-CS Neg**, N = 8,695	p-value[1]
Ferritin			<0.001
Median (IQR)	3,490 (1,886, 5,927)	601 (246, 1,221)	
Unknown	1 (0.3%)	7,601 (87%)	
IL_6			<0.001
Median (IQR)	39 (16, 116)	20 (10, 48)	
Unknown	23 (6.8%)	8,325 (96%)	
CRP			<0.001
Median (IQR)	146 (95, 225)	62 (14, 135)	
Unknown	1 (0.3%)	7,494 (86%)	
ALT			<0.001
Median (IQR)	45 (27, 85)	24 (16, 40)	
Unknown	1 (0.3%)	5,626 (65%)	
Alk_Phos			<0.001
Median (IQR)	114 (86, 184)	93 (73, 125)	
Unknown	1 (0.3%)	5,671 (65%)	
Bili_Direct			<0.001
Median (IQR)	0.30 (0.20, 0.60)	0.20 (0.20, 0.30)	
Unknown	68 (20%)	7,528 (87%)	
Bili_Indirect			<0.001
Median (IQR)	0.60 (0.40, 0.90)	0.50 (0.40, 0.70)	
Unknown	68 (20%)	7,529 (87%)	
Bili_Total			<0.001
Median (IQR)	0.70 (0.50, 1.23)	0.50 (0.40, 0.70)	
Unknown	9 (2.7%)	6,016 (69%)	
Creatinine			<0.001
Median (IQR)	1.13 (0.85, 1.65)	0.93 (0.75, 1.17)	
Unknown	0 (0%)	5,463 (63%)	
D.Dimer			<0.001
Median (IQR)	2.8 (1.5, 7.3)	1.4 (0.7, 3.1)	
Unknown	22 (6.5%)	7,625 (88%)	
Fibrinogen			<0.001
Median (IQR)	335 (232, 450)	401 (316, 499)	
Unknown	20 (5.9%)	7,688 (88%)	
IL_2_Recp			0.9
Median (IQR)	2,609 (1,465, 7,628)	4,206 (1,028, 10,673)	
Unknown	319 (95%)	8,689 (100%)	
LDH			<0.001

© 2025 Long JP et al. *JAMA Network Open*.

**Characteristic**	**COVID-CS**, N = 337	**COVID-CS Neg**, N = 8,695	**p-value**[1]
Median (IQR)	429 (292, 656)	247 (197, 340)	
Unknown	3 (0.9%)	6,700 (77%)	
Lymphocyte_Abs			<0.001
Median (IQR)	1.02 (0.58, 1.69)	1.29 (0.85, 1.85)	
Unknown	3 (0.9%)	5,426 (62%)	
Monocyte_Abs			<0.001
Median (IQR)	0.74 (0.45, 1.28)	0.60 (0.45, 0.84)	
Unknown	3 (0.9%)	5,433 (62%)	
Neutrophil_Abs			<0.001
Median (IQR)	6.7 (4.1, 12.8)	4.2 (2.8, 6.6)	
Unknown	3 (0.9%)	5,426 (62%)	
Platelet_count			<0.001
Median (IQR)	65 (20, 128)	186 (125, 245)	
Unknown	1 (0.3%)	5,377 (62%)	
TNF_alpha			<0.001
Median (IQR)	25 (17, 44)	17 (11, 25)	
Unknown	121 (36%)	8,367 (96%)	
Troponin_T			<0.001
Median (IQR)	30 (16, 61)	18 (11, 32)	
Unknown	50 (15%)	7,800 (90%)	
V_Lactate			<0.001
Median (IQR)	1.60 (1.10, 2.50)	1.30 (0.90, 1.70)	
Unknown	151 (45%)	8,192 (94%)	
WBC			<0.001
Median (IQR)	2.30 (1.00, 4.00)	4.90 (3.30, 6.70)	
Unknown	0 (0%)	5,392 (62%)	

[1]Wilcoxon rank sum test; Wilcoxon rank sum exact test

© 2025 Long JP et al. *JAMA Network Open*.

eTable 2: Summary of missingness

Characteristic	N = 671[1]
WBC	0 (0%)
Neutrophil_Abs	29 (4.3%)
Lymphocyte_Abs	31 (4.6%)
Monocyte_Abs	33 (4.9%)
Hgb	0 (0%)
Platelet_count	0 (0%)
Fibrinogen	47 (7.0%)
D.Dimer	109 (16%)
CRP	74 (11%)
ESR	404 (60%)
Ferritin	7 (1.0%)
LDH	1 (0.1%)
IL_6	342 (51%)
IL_2_Recp	442 (66%)
TNF_alpha	458 (68%)
ALT	1 (0.1%)
Alk_Phos	1 (0.1%)
Bili_Total	3 (0.4%)
Bili_Direct	76 (11%)
Bili_Indirect	76 (11%)
Albumin_Lvl	1 (0.1%)
Creatinine	1 (0.1%)
Troponin_T	330 (49%)
Sodium_Lvl	0 (0%)
V_Lactate	357 (53%)

[1]n (%)

© 2025 Long JP et al. *JAMA Network Open*.

eTable 3. Regression model (controlled for the effect of race, gender, and age) of laboratory values compared by cause of CS (COVID-CS, CAR-T CRS, and MA-HLH).

Lab	Covid-CS vs. CAR-T CRS	MA-HLH vs. CAR-T CRS	MA-HLH vs. Covid-CS
**D.Dimer**	<0.001	<0.001	<0.001
**Albumin_Lvl**	0.004	<0.001	<0.001
**Ferritin**	<0.001	<0.001	<0.001
**Hgb**	0.312	<0.001	<0.001
**CRP**	0.091	0.682	0.274
**IL_6**	0.092	0.086	<0.001
**WBC**	<0.001	0.001	<0.001
**Fibrinogen**	<0.001	<0.001	<0.001
**TNF_alpha**	0.017	<0.001	<0.001

© 2025 Long JP et al. *JAMA Network Open*.

eTable 4. Cox proportional hazards model regressing survival on demographic variables, and cohort.

Characteristic	HR[1]	95% CI[1]	p-value
**Cohort**			
**CAR-T CRS**	—	—	
**COVID-CS**	2.93	1.95, 4.41	<0.001
**MA-HLH**	8.12	5.51, 12.0	<0.001
**Race**			
**Caucasian**	—	—	
**Other**	1.12	0.80, 1.56	0.5
**Black or AA**	0.84	0.59, 1.21	0.4
**Asian**	1.13	0.67, 1.89	0.6
**Gender**			
**F**	—	—	
**M**	0.93	0.73, 1.18	0.5
**Age**	1.01	1.01, 1.02	<0.001

[1]HR = Hazard Ratio, CI = Confidence Interval

© 2025 Long JP et al. *JAMA Network Open*.

eTable 5. Univariate Cox PH of laboratory values comparing CAR-T CRS, COVID-CS, and MA-HLH cohorts.

Characteristic	N	HR[1]	95% CI[1]	p-value
**Gender**	671			
**F**		—	—	
**M**		0.79	0.63, 1.00	0.054
**Race**	667			
**Caucasian**		—	—	
**Other**		1.14	0.82, 1.59	0.435
**Black or AA**		0.94	0.66, 1.36	0.758
**Asian**		1.31	0.79, 2.18	0.302
**Age**	671			
**(0,50]**		—	—	
**(50,60]**		0.97	0.70, 1.34	0.852
**(60,70]**		0.79	0.58, 1.07	0.128
**(70,120]**		0.95	0.69, 1.32	0.764
**WBC**	671	0.73	0.64, 0.83	<0.001
**Neutrophil_Abs**	642	0.78	0.69, 0.89	<0.001
**Lymphocyte_Abs**	640	0.87	0.76, 0.99	0.032
**Monocyte_Abs**	638	0.77	0.68, 0.88	<0.001
**Hgb**	671	0.57	0.51, 0.64	<0.001
**Platelet_count**	671	0.48	0.42, 0.54	<0.001
**Fibrinogen**	624	0.67	0.60, 0.76	<0.001
**D.Dimer**	562	1.92	1.69, 2.19	<0.001
**CRP**	597	1.30	1.14, 1.48	<0.001
**ESR**	267	1.02	0.86, 1.21	0.838
**Ferritin**	664	2.45	2.16, 2.79	<0.001
**LDH**	670	2.24	1.97, 2.55	<0.001
**IL_6**	329	1.69	1.42, 2.00	<0.001
**IL_2_Recp**	229	1.24	1.06, 1.44	0.006
**TNF_alpha**	213	1.46	1.19, 1.80	<0.001
**ALT**	670	1.44	1.27, 1.62	<0.001
**Alk_Phos**	670	1.85	1.64, 2.08	<0.001
**Bili_Total**	668	1.96	1.72, 2.22	<0.001
**Bili_Direct**	595	2.12	1.86, 2.40	<0.001
**Bili_Indirect**	595	1.49	1.31, 1.69	<0.001
**Albumin_Lvl**	670	0.58	0.52, 0.65	<0.001
**Creatinine**	670	1.34	1.20, 1.51	<0.001
**Troponin_T**	341	1.51	1.30, 1.75	<0.001

© 2025 Long JP et al. *JAMA Network Open*.

Characteristic	N	HR[1]	95% CI[1]	p-value
**Sodium_Lvl**	671	0.87	0.77, 0.98	0.018
**V_Lactate**	314	1.71	1.43, 2.04	<0.001
**Cohort**	671			
**CAR-T CRS**		—	—	
**COVID-CS**		2.92	1.94, 4.37	<0.001
**MA-HLH**		7.20	4.93, 10.5	<0.001

[1]HR = Hazard Ratio, CI = Confidence Interval. Abbreviations: Hazard ratios (HR), 95% confidence intervals (CI), and p-values.

© 2025 Long JP et al. *JAMA Network Open*.

eTable 6. Multivariate Cox PH of laboratory values comparing CAR-T CRS, COVID-CS, and MA-HLH cohorts.

Characteristic	HR[1]	95% CI[1]	p-value
**Gender**			
**F**	—	—	
**M**	0.82	0.63, 1.06	0.13
**Race**			
**Caucasian**	—	—	
**Other**	1.12	0.79, 1.60	0.5
**Black or AA**	0.82	0.56, 1.20	0.3
**Asian**	1.48	0.86, 2.55	0.2
**Age**			
**(0,50]**	—	—	
**(50,60]**	1.35	0.96, 1.90	0.088
**(60,70]**	1.14	0.82, 1.60	0.4
**(70,120]**	1.75	1.21, 2.54	0.003
**WBC**	1.24	1.04, 1.48	0.016
**Neutrophil_Abs**	0.86	0.72, 1.02	0.088
**Lymphocyte_Abs**	0.99	0.84, 1.17	>0.9
**Monocyte_Abs**	0.98	0.81, 1.19	0.8
**Hgb**	1.13	0.94, 1.37	0.2
**Platelet_count**	0.57	0.47, 0.70	<0.001
**Fibrinogen**	0.95	0.82, 1.10	0.5
**D.Dimer**	1.04	0.88, 1.24	0.6
**CRP**	1.16	1.01, 1.35	0.038
**ESR**	0.91	0.73, 1.13	0.4
**Ferritin**	1.30	1.03, 1.63	0.025
**LDH**	1.36	1.13, 1.63	0.001
**IL_6**	1.38	1.13, 1.67	0.002
**IL_2_Recp**	1.09	0.90, 1.31	0.4
**TNF_alpha**	0.83	0.66, 1.06	0.13
**ALT**	0.85	0.72, 1.00	0.052
**Alk_Phos**	1.15	0.97, 1.36	0.11
**Bili_Total**	0.95	0.71, 1.26	0.7
**Bili_Direct**	1.38	1.02, 1.87	0.037
**Bili_Indirect**	0.98	0.79, 1.22	0.9
**Albumin_Lvl**	0.92	0.77, 1.09	0.3
**Creatinine**	1.06	0.93, 1.21	0.4
**Troponin_T**	1.16	0.96, 1.41	0.13

© 2025 Long JP et al. *JAMA Network Open*.

Characteristic	HR[1]	95% CI[1]	p-value
**Sodium_Lvl**	1.20	1.06, 1.35	0.003
**V_Lactate**	1.08	0.88, 1.32	0.5
**Cohort**			
**CAR-T CRS**	—	—	
**COVID-CS**	2.61	1.65, 4.12	<0.001
**MA-HLH**	2.55	1.57, 4.13	<0.001

[1]HR = Hazard Ratio, CI = Confidence Interval. Abbreviations: Hazard ratios (HR), 95% confidence intervals (CI), and p-values.

© 2025 Long JP et al. *JAMA Network Open*.

eFigure 1: Flow Diagram

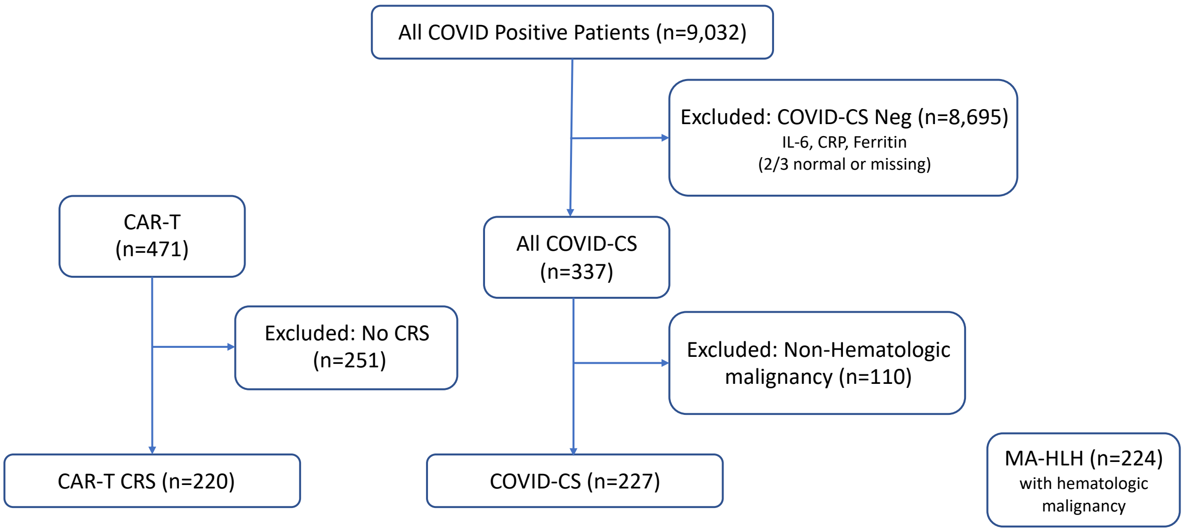

© 2025 Long JP et al. *JAMA Network Open*.

eFigure 2: Comparison of survival between COVID-19 patients diagnosed with Cytokine Storm (COVID-CS) and COVID-19 patients not diagnosed with cytokine storm (COVID-CS Neg).

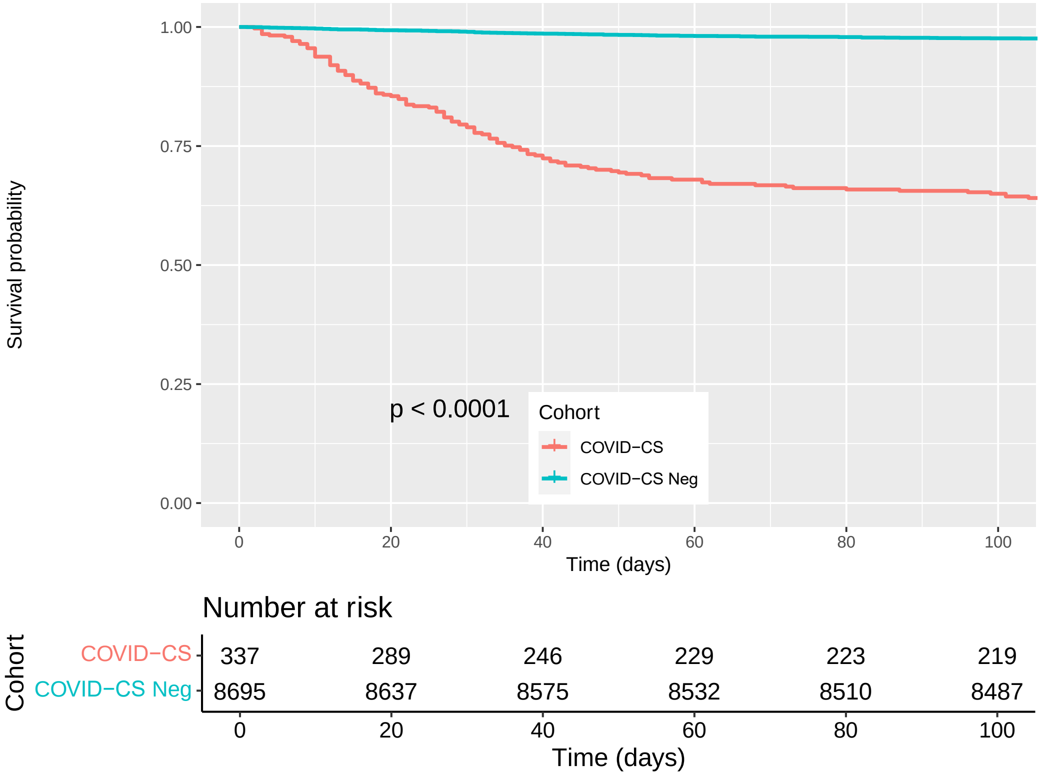

© 2025 Long JP et al. *JAMA Network Open*.

eFigure 3: Association of tocilizumab with overall survival by cohort.

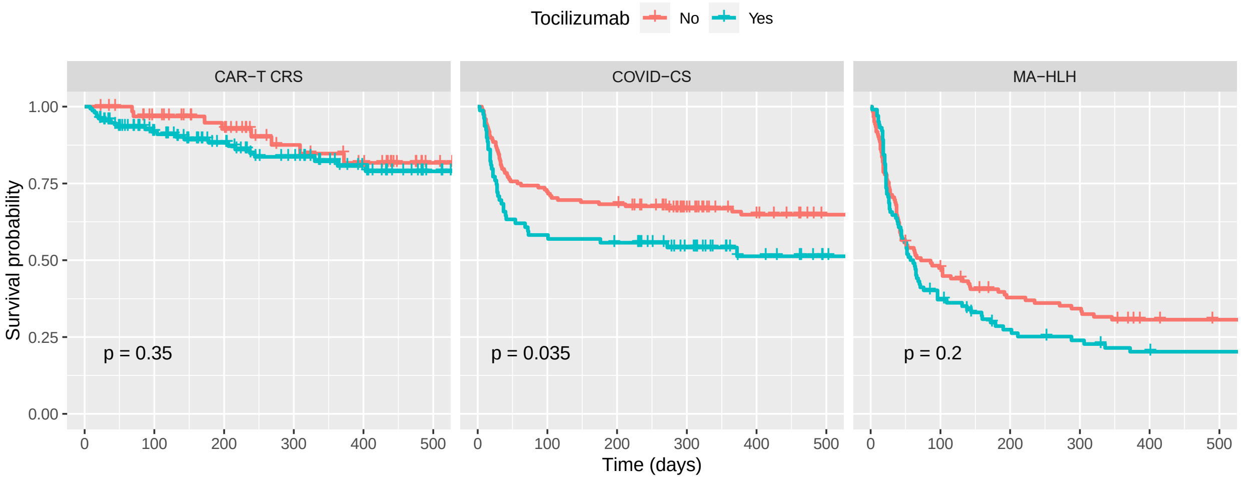

© 2025 Long JP et al. *JAMA Network Open*.

eFigure 4: Variable importance in the Random Survival Forest (RSF) model. Higher variable importance implies the variable has a larger impact on prediction performance.

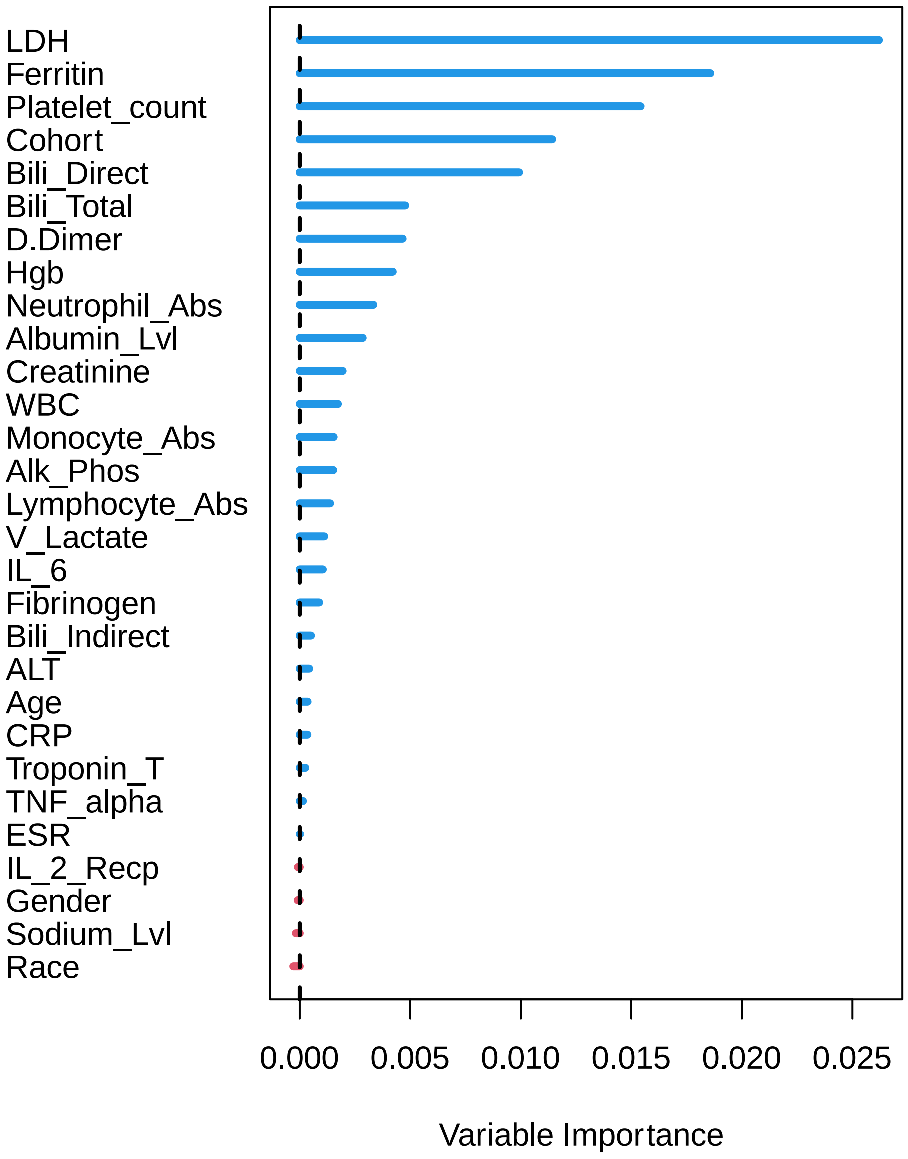

© 2025 Long JP et al. *JAMA Network Open*.
