# Supplementary material for: Cytokine Storms in COVID-19, Hemophagocytic Lymphohistiocytosis, and CAR-T Therapy
Source: JAMA Netw Open. 2025 Apr 7;8(4):e253455. doi: 10.1001/jamanetworkopen.2025.3455 (PMC11976493; doi:10.1001/jamanetworkopen.2025.3455)
Supplement: Supplement 2. — Data-Driven Determinants for COVID-19 Discovery Effort (D3CODE) Team [file jamanetwopen-e253455-s002.pdf]

Supplemental Online Content: Nonauthor Collaborators
*First name, last name, and suffix (if applicable) are required and will appear in PubMed.

*Group Name(s): Data-Driven Determinants for COVID-19 Discovery Effort (D3CODE) Team							
***First Name and Middle Initial(s)**	***Last Name**	***Suffix (eg, Jr, III)**	Academic Degrees	Institution	Location (city, state/province, country)	Role or Contribution, eg, chair, principal investigator	Group (if more than 1 Group listed in the byline) and/or Subgroup (eg, Steering Committee)
Aaroe	Ashley			The University of Texas MD Anderson Cancer Center, Houston TX, USA			
Abraham	Sanu			The University of Texas MD Anderson Cancer Center, Houston TX, USA			
Aloia	Thomas A			Ascension, St. Louis, MO, USA			
Andrews	Lee	II		The University of Texas MD Anderson Cancer Center, Houston TX, USA			
Badami	Kiran K			The University of Texas MD Anderson Cancer Center, Houston TX, USA			
Baganz	Janna A			The University of Texas MD Anderson Cancer Center, Houston TX, USA			
Bajwa	Pratibha			The University of Texas MD Anderson Cancer Center, Houston TX, USA			
Barbosa	Gregory R			The University of Texas MD Anderson Cancer Center, Houston TX, USA			
Beird	Hannah C			The University of Texas MD Anderson Cancer Center, Houston TX, USA			
Brock	Kristy			The University of Texas MD Anderson Cancer Center, Houston TX, USA			
Burton	Elizabeth M			The University of Texas MD Anderson Cancer Center, Houston TX, USA			
Cata	Juan			The University of Texas MD Anderson Cancer Center, Houston TX, USA			
Chung	Caroline			The University of Texas MD Anderson Cancer Center, Houston TX, USA			
Claussen	Catherine M			The University of Texas MD Anderson Cancer Center, Houston TX, USA			
Crommett	John			The University of Texas MD Anderson Cancer Center, Houston TX, USA			
Cuenca Trujillo	John			The University of Texas MD Anderson Cancer Center, Houston TX, USA			
Cutherell	Michael			The University of Texas MD Anderson Cancer Center, Houston TX, USA			
Dabaja	Bouthaina			The University of Texas MD Anderson Cancer Center, Houston TX, USA			
Dagher	Hiba			The University of Texas MD Anderson Cancer Center, Houston TX, USA			
Daniels	Kevin M			The University of Texas MD Anderson Cancer Center, Houston TX, USA			
Domask	Mary			The University of Texas MD Anderson Cancer Center, Houston TX, USA			
Draetta	Giulio			The University of Texas MD Anderson Cancer Center, Houston TX, USA			
Edelkamp	Paul	Jr		The University of Texas MD Anderson Cancer Center, Houston TX, USA			
Fisher	Sarah			The University of Texas MD Anderson Cancer Center, Houston TX, USA			
French	Katy E			The University of Texas MD Anderson Cancer Center, Houston TX, USA			
Futreal	Andrew			The University of Texas MD Anderson Cancer Center, Houston TX, USA			
Gaeta	Maria			The University of Texas MD Anderson Cancer Center, Houston TX, USA			
Gibbons	Christopher			Oracle Health, Kansas City, MO, USA			
Godoy	Myrna			The University of Texas MD Anderson Cancer Center, Houston TX, USA			

Supplemental Online Content: Nonauthor Collaborators
*First name, last name, and suffix (if applicable) are required and will appear in PubMed.

***First Name and Middle Initial(s)**	***Last Name**	***Suffix (eg, Jr, III)**	Academic Degrees	Institution	Location (city, state/province, country)	Role or Contribution, eg, chair, principal investigator	Group (if more than 1 Group listed in the byline) and/or Subgroup (eg, Steering Committee)
Goldstein	Drew			The University of Texas MD Anderson Cancer Center, Houston TX, USA			
Gunther	Jillian			The University of Texas MD Anderson Cancer Center, Houston TX, USA			
Hutcheson	Kate			The University of Texas MD Anderson Cancer Center, Houston TX, USA			
Jaffray	David			The University of Texas MD Anderson Cancer Center, Houston TX, USA			
Jin	Jeff			The University of Texas MD Anderson Cancer Center, Houston TX, USA			
John	Teny M			The University of Texas MD Anderson Cancer Center, Houston TX, USA			
Kell	Trey			The University of Texas MD Anderson Cancer Center, Houston TX, USA			
Knafl	Mark			The University of Texas MD Anderson Cancer Center, Houston TX, USA			
Kothari	Anai			Medical College of Wisconson, Milwauke Wisconsin, USA			
Kwan	Rayson C			The University of Texas MD Anderson Cancer Center, Houston TX, USA			
Lee	J.Jack			The University of Texas MD Anderson Cancer Center, Houston TX, USA			
Litton	Jennifer			The University of Texas MD Anderson Cancer Center, Houston TX, USA			
McEnery	Kevin W			The University of Texas MD Anderson Cancer Center, Houston TX, USA			
McGuire	Mary			The University of Texas MD Anderson Cancer Center, Houston TX, USA			
Mescher	Benjamin			The University of Texas MD Anderson Cancer Center, Houston TX, USA			
Musunuru	Tejo			The University of Texas MD Anderson Cancer Center, Houston TX, USA			
Muthu	Mayoora			The University of Texas MD Anderson Cancer Center, Houston TX, USA			
Nates	Joseph			The University of Texas MD Anderson Cancer Center, Houston TX, USA			
Owen	Craig S			The University of Texas MD Anderson Cancer Center, Houston TX, USA			
Padmakumar	Priyadharshini			The University of Texas MD Anderson Cancer Center, Houston TX, USA			
Palaskas	Nicholas			The University of Texas MD Anderson Cancer Center, Houston TX, USA			
Patel	Jay J			The University of Texas MD Anderson Cancer Center, Houston TX, USA			
Prabhakaran	Sabitha			The University of Texas MD Anderson Cancer Center, Houston TX, USA			
Ramsey	Lucas			The University of Texas MD Anderson Cancer Center, Houston TX, USA			
Ravi	Vinod			The University of Texas MD Anderson Cancer Center, Houston TX, USA			
Rojas Hernandez	Cristhiam			The University of Texas MD Anderson Cancer Center, Houston TX, USA			
Sajith	Bilja			The University of Texas MD Anderson Cancer Center, Houston TX, USA			
Scheet	Paul A			The University of Texas MD Anderson Cancer Center, Houston TX, USA			
Schmidt	Stephanie			The University of Texas MD Anderson Cancer Center, Houston TX, USA			
Shaw	Kenna R			The University of Texas MD Anderson Cancer Center, Houston TX, USA			
Shete	Sanjay			The University of Texas MD Anderson Cancer Center, Houston TX, USA			

Supplemental Online Content: Nonauthor Collaborators
*First name, last name, and suffix (if applicable) are required and will appear in PubMed.

***First Name and Middle Initial(s)**	***Last Name**	***Suffix (eg, Jr, III)**	Academic Degrees	Institution	Location (city, state/province, country)	Role or Contribution, eg, chair, principal investigator	Group (if more than 1 Group listed in the byline) and/or Subgroup (eg, Steering Committee)
Shoenthal	Daniel P			The University of Texas MD Anderson Cancer Center, Houston TX, USA			
Stoltenberg	Lessley J			The University of Texas MD Anderson Cancer Center, Houston TX, USA			
Subbiah	Ishwaria			Sarah Cannon Research Institute, Nashville Tenesse, USA			
Tawbi	Hussein			The University of Texas MD Anderson Cancer Center, Houston TX, USA			
Thompson	Phillip			Monash Institute of Pharmaceutical Sciences, Monash University, Parkville Victoria, Australia			
Turin	Anastasia			The University of Texas MD Anderson Cancer Center, Houston TX, USA			
Unni	Samir			The University of Texas MD Anderson Cancer Center, Houston TX, USA			
Vicknamparampil	Benju			The University of Texas MD Anderson Cancer Center, Houston TX, USA			
Weber	Max C			The University of Texas MD Anderson Cancer Center, Houston TX, USA			
Weinstein	John			The University of Texas MD Anderson Cancer Center, Houston TX, USA			
Woodman	Scott Eric			The University of Texas MD Anderson Cancer Center, Houston TX, USA			
Wozny	Mark C			The University of Texas MD Anderson Cancer Center, Houston TX, USA			
Wu	Carol			The University of Texas MD Anderson Cancer Center, Houston TX, USA			
Wu	Jia			The University of Texas MD Anderson Cancer Center, Houston TX, USA			
Yao	James C			The University of Texas MD Anderson Cancer Center, Houston TX, USA			
Young	Chingyi			The University of Texas MD Anderson Cancer Center, Houston TX, USA			
Yu	Emily			The University of Texas MD Anderson Cancer Center, Houston TX, USA			
Zatorski	Steven			The University of Texas MD Anderson Cancer Center, Houston TX, USA			
