# Supplementary material for: Cytokine Storms in COVID-19, Hemophagocytic Lymphohistiocytosis, and CAR-T Therapy
Source: JAMA Netw Open. 2025 Apr 7;8(4):e253455. doi: 10.1001/jamanetworkopen.2025.3455 (PMC11976493; doi:10.1001/jamanetworkopen.2025.3455)
Supplement: Supplement 3. — Data Sharing Statement [file jamanetwopen-e253455-s003.pdf]

Data Sharing Statement

Long. Cytokine Storms in COVID-19, Hemophagocytic Lymphohistiocytosis, and CAR-T Therapy. *JAMA Netw Open*. Published April 07, 2025.
doi:10.1001/jamanetworkopen.2025.3455

Data
Data available: No

Additional Information
Explanation for why data not available: Health informatics data comes from a proprietary data extraction SaaS platform. Curated unidentified data can be made available on reasonable request
